# Supplementary material for: Carbonic Anhydrase Inhibition as a Target for Antibiotic Synergy in Enterococci
Source: Microbiol Spectr. 2023 Jun 1;11(4):e03963-22. doi: 10.1128/spectrum.03963-22 (PMC10434275; doi:10.1128/spectrum.03963-22)
Supplement: Supplemental file 1 — Fig. S1. Download spectrum.03963-22-s0001.pdf, PDF file, 0.06 MB [file spectrum.03963-22-s0001.pdf]

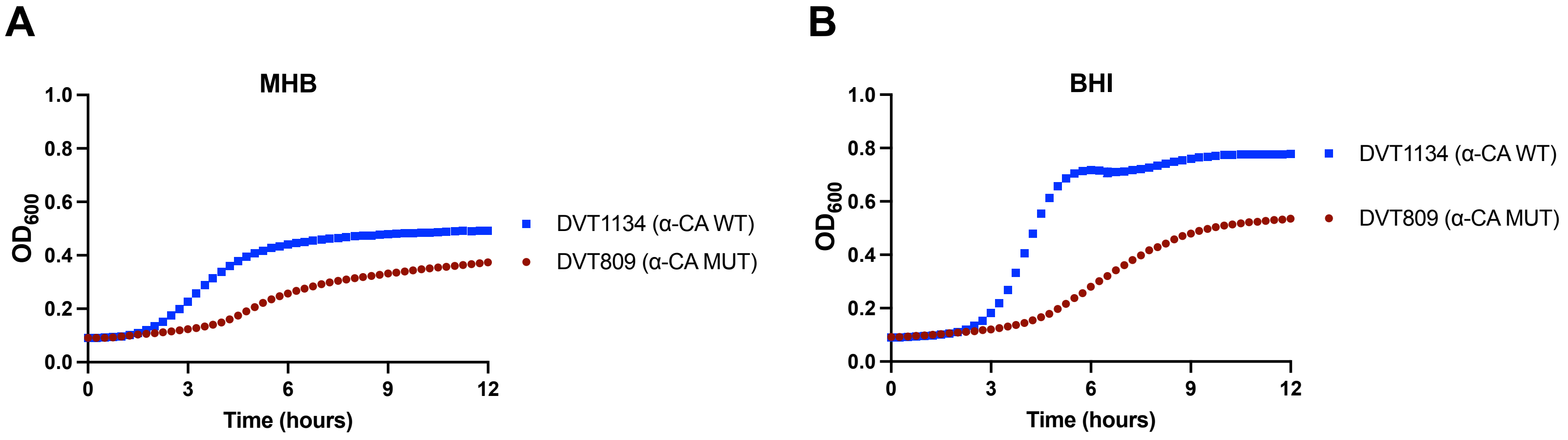

FIG S1. *In vitro* growth differences between wild-type (WT) and alpha-carbonic anhydrase (α-CA) mutant (MUT) *E. faecalis* strains. Strains were inoculated from overnight cultures into (A) Mueller Hinton Broth (MHB) or (B) Brain Heart Infusion (BHI) media and were grown at 37°C. OD600 was recorded every 30 minutes for 12 hours.
